# Phonological iconicity

**DOI:** 10.3389/fpsyg.2014.00080

**Published:** 2014-02-12

**Authors:** David S. Schmidtke, Markus Conrad, Arthur M. Jacobs

**Affiliations:** ^1^Department of General Psychology and Neurocognitive, Freie Universität BerlinBerlin, Germany; ^2^Department of Cognitive Neuroscience and Psycholinguistics, Universidad de La LagunaLa Laguna, Spain; ^3^Dahlem Institute for Neuroimaging of Emotion (DINE)Berlin, Germany

**Keywords:** phonological iconicity, sound symbolism, phonaesthemes, kiki/bouba effect, ideophones

## Abstract

The arbitrariness of the linguistic sign is a fundamental assumption in modern linguistic theory. In recent years, however, a growing amount of research has investigated the nature of non-arbitrary relations between linguistic sounds and semantics. This review aims at illustrating the amount of findings obtained so far and to organize and evaluate different lines of research dedicated to the issue of phonological iconicity. In particular, we summarize findings on the processing of onomatopoetic expressions, ideophones, and phonaesthemes, relations between syntactic classes and phonology, as well as sound-shape and sound-affect correspondences at the level of phonemic contrasts. Many of these findings have been obtained across a range of different languages suggesting an internal relation between sublexical units and attributes as a potentially universal pattern.

## Introduction

Linguistic theory widely adopts Saussure's (1959) essential notion of an arbitrary relation between signifier and signified. While exceptions to this rule have been suggested outside the linguistic mainstream (Jakobson and Waugh, 1979; Tsur, 1992, 1997; Hinton et al., 1994; Volke, 2007; Schrott and Jacobs, 2011), most psycholinguistic models of lexical retrieval and production (e.g., Dell and O'Seaghdha, 1992; Levelt et al., 1999) incorporate arbitrariness as a fundamental feature. However, recent research posits motivated sound-meaning mappings (see Perniss et al., 2010, for review), that according to Peirce's prolific typology of semiotic elements (Peirce, 1931; see Liszka, 1996 for an overview) classify as iconic or indexical rather than symbolic, involving structural resemblance, or natural association between signifier and signified.

Empirical evidence for such phenomena primarily comes from signed languages (e.g., Thompson et al., 2012), gesture (e.g., McNeill, 2008), or prosody (e.g., Nygaard et al., 2009b). Evidence in phonology in spoken languages is, though, less determined and will be outlined subsequently regarding the role of iconicity as pivotal in human language.

We will first focus on onomatopoiea and ideophones as well-established sound-symbolic inventories in a variety of languages. Phoneasthemes intoduce the basic idea of sublexical units referring to higher level attributes of meaning, giving rise to different approaches particularly concerning the phonemic level in relation to affect or the perception of size or shape. We thus aim to interrogate the nature of iconicity in language processing and its role in phylogenetic and ontogenetic language development.

## Onomatopoeia

Intuitively, phonological iconicity is reflected in onomatopoeia that mimic animal sounds or sounds habitually associated with moving or colliding objects (e.g., *cuckoo*, *bang*) sometimes further imitating the emotional impression they have on us, e.g., the German “*Uff*” which transposes the ejected breath (*ff*) with which we instinctively express a reaction of relief into written German (Schrott and Jacobs, 2011). According to Berko-Gleason (2005), word acquisition in early childhood often refers to onomatopoeic expressions, because their inherent echoic relation to a referent enhances apprehension (cf. also Perniss and Vigliocco, in press).

Using functional magnetic resonance imaging, Hashimoto et al. (2006) reported that nouns increased activation in the left anterior superior temporal gyrus and animal sounds in the bilateral superior temporal sulcus and the left inferior frontal gyrus, while onomatopoeia recruited structures involved in the processing of both, thus indicating the activation of neural subsystems devoted to perception beyond language comprehension only.

According to Wundt (1904), some onomatopoeia occur as interjections, i.e., non-sentence phrases expressing emotion or sentiment on the speaker's part (e.g., *Ah!*, *Pst!*).

Following Schrott and Jacobs (2011), in interjections language seems closest to (affective) mental life (cf. also Bühler, 1934; Wierzbicka, 1991). They lend a voice to bodily feelings and affects, e.g., pain (German “aua”) or indifference (German “bah”). The Yiddish interjection “oy” expresses no less than 29 different affect states in only two phonemes (Rosten, 1968). Reaching beyond the expressive function in Bühler's (1934) *Organon model*, they also fulfill the conative/appealing function as in the calling (German “he”) or the request to keep silent (German “ssst”).

Testing cross-cultural agreement in the understanding of phonological iconicity of interjections, Sauter et al. (2010) asked native English speakers and speakers of Himba, a Namibian Bantu language, which of two vocalizations of the respective unknown language would best match a presented short story. Though participants agreed cross-culturally, the question remains whether they inferred the correct meaning from phonologic stimulus features or other acoustic cues, as Couper-Kuhlen (2011) demonstrated that the interpretation of “oh” as utterance of disappointment or anger much depends on prosody modulated by volume, pitch and intonation.

## Ideophones, mimetics, expressives

Ideophones, mimetics, or expressives, typically referring to sound-symbolic inventories of Sub Saharan African, East Asian or Native American languages, similarly elude standard linguistic theory. According to Dingemanse (2011, 2012), they “depict sensory imagery” rather than merely describing it, and reach, unlike onomatopoeia, beyond acoustic perception only (e.g., Japanese *kyoro kyoro* for “looking around” or “spinning”; Tamil *thuru thuru* for “eager” or “active”). Following Dingemanse, sensory imagery is perceptual knowledge that derives from sensory perception of the environment and the body. Although scarcely represented in Indo-European languages, Atoda and Hoshino (1995) list more than 1700 frequent Japanese mimetic words, thus exceeding onomatopoiea numerically.

Iwasaki et al. (2007) showed that Japanese and English monolinguals agree in evaluative ratings of Japanese ideophones, despite Japanese raters' higher degrees of consistency. Effects were stronger for concepts of sound than vision or proprioception and limited to certain phonemes, but still suggest certain sound-meaning mappings to generalize cross-linguistically, which cannot be explained by mere exposure to language regularities.

Imai et al. (2008) replicated this result with ideophonic neologisms in Japanese and English native speakers. Using the same stimuli in a subsequent verb learning task with 3-year-old Japanese children, they further demonstrated that ideophonic word material facilitates verb acquisition in toddlers—predominantly due to phonological as opposed to morphological or syntactic properties. Kantartzis et al. (2011) and Yoshida (2012) extended these findings to English children creating comparable complements despite the marginal incidence of ideophones in their native language.

Using a word learning task, Nygaard et al. (2009a) reported higher accuracy and faster responses of English speaking monolingual adults to correct translations of Japanese adjectives involving a variety of perceptuo-motor properties. The effect was even present when matched to their antonyms—though to a lesser extent—as compared to random assignments. Iconic mappings thus reach beyond acoustic experience and hold across unrelated languages.

## Lexical categories

Focusing on broader syntactic categories rather than distinct attributes grounded in sensory domains, effects of regular phonological mappings are abundant also in Indo-European languages. Nouns are likely to count more syllables than verbs (Cassidy and Kelly, 1991) or to contain back (e.g., /u/,/o/) rather than front vowels (e.g., /e/,/i/) (Kelly, 1992). Nouns and verbs also exhibit larger Euclidean phonological distances across word classes than within (Farmer et al., 2006). English female names differ from male names and other nouns in number of syllables, syllable stress, and vowel brightness (Cutler et al., 1990). More importantly, language users exploit these regularities during language development when learning to assign new words to grammatical classes (Cassidy et al., 1999; Cassidy and Kelly, 2001; Farmer et al., 2006; Reilly et al., 2012).

These results imply systematic relations between phonology and syntax, rather than semantics. Yet, from a connectionist perspective, morphology might emerge as a layer of hidden units between levels of phonology and semantics (Plaut and Gonnerman, 2000). Accordingly, Monaghan et al. (2011) point out that morphology generates numerous instances of systematicity serving category assignment in first language acquisition, some of which (e.g., plural forms or differences in female vs. male names) might be considered iconic.

## Phonaesthemes

These are phoneme clusters like syllable onsets or rimes that typically occur in words belonging to specific semantic fields, (e.g., *gl*, as in *glitter, glow, gleam* etc. relates *to “vision”* and *“light”*) but lack the central feature of compositionality to qualify as morphemes. They even appear across language borders in non-cognate-words of remote languages (e.g., the consonant sequence /s/t/r/ reflecting concepts of “straight” in both English and Gaelic, Magnus, 2000). Several studies in English and Swedish posit phonaesthemes as instrumental in production and perception of neologisms (Hutchins, 1998; Abelin, 1999; Magnus, 2000). Bergen (2004) reported priming effects for phonaesthemic prime-target relations to be more pronounced than predicted by linearly combined effects of phonological and semantic priming. In a word learning task, phonaesthemes facilitated participants' deduction of new meanings with or without context (Parault, 2006).

According to Bergen (2004), available data do not necessarily suggest an innate sound-meaning relation. They might well be accounted for by connectionist models in terms of acquired associative frequency effects (e.g., Grainger and Jacobs, 1996; Rey et al., 1998; Plaut and Gonnerman, 2000), and were also suggested to have derived from early indo-european morphemes indicating etymologic evolution rather than iconic relation to referents as source of their occurrence. Note, however, that specific phonaesthemes such as *sn*—involving a nasal sound—occurring in words related to the nose (*sniff, snore, snob*) also seem to depict sensory imagery and therefore might qualify as iconic mappings.

## Phonemic contrasts

### Sound and size

Sapir (1929) initiated an influential line of research focusing on phonemic contrasts. Using nonword pairs, thus addressing potential sound-meaning mappings beyond the direct context of a given vocabulary, he showed that English speakers systematically associate the back vowel /a/ with largeness, but the front vowel /i/ with smallness. Newman (1933) extended his finding showing that size judgments systematically co-vary with articulation point in the vocal tract for consonants and vowels—more frontal phonemes relate to smallness and vice versa, yet failed to establish such sound-size relations for 350 English words with size connotations. Using alternative methods, Taylor and Taylor (1965) were able to reveal statistically reliable relations within Newman's data of smallness with more frontal sounds (e.g., consonants /n/,/t/; vowels /e/,/i/) as well as largeness with more posterior sounds (e.g., /g/,/k/; /o/,/u/).

More recently, Peña et al. (2011) reported increased looking times of 4-month-old infants for front vowels (/e/,/i/) presented with smaller, and back vowels (/a/,/o/) presented with larger objects than vice versa. Using a broader range of phonologically comparable nonword stimuli, Thompson and Estes (2011) demonstrated that this effect follows a graded function in adults. They argue that cross-modal processing of gesture and frequency code (Ohala, 1982; Berlin, 2006) better account for the results than statistical learning. In his frequency code hypothesis, Ohala (1984) stresses the correlation between general physical and vocal tract size: the fundamental frequency modulation (F0) would be the acoustic counterpart of common visual displays of physical size, providing a close link to natural selection—a pattern that might reverberate in the perception of vowel backness.

Shrum et al. (2012) extended empirical findings cross-linguistically: across French, Spanish, and Chinese subjects, fictitious brand names were preferred when vowel backness matched products' perceived size attributes.

### Sound and shape

Substantial evidence for phonological iconicity as a cross-linguistic phenomenon was derived from a seminal experiment of Köhler (1929). Within the framework of Gestalt psychology, he showed a reliable preference of native Spanish speakers to match the nonword *maluma* with a curvy round shape and *takete* with a spiky angular shape. The effect was subsequently labeled as “kiki/bouba effect” and replicated across a wide range of unrelated languages such as Himba (Bremner et al., 2012) or Tamil. It appears to be extraordinarily reliable with agreement of up to 95% (Ramachandran and Hubbard, 2001).

Maurer et al. (2006) found this effect in 2.5-year-old preliterate toddlers using a forced choice task. Ozturk et al. (2012) even demonstrated effects of congruent vs. incongruent sound-shape mappings in looking times of 4-month-old children. Infants' attention differed significantly though exclusively to a combination of continuants (e.g., /b/) and back vowels (e.g., /u/) or plosives (e.g., /k/) and front vowels (e.g., /i/), respectively. Adults' judgments from a control study revealed sensitivity to consonants or vowels only.

Developmental and cross-linguistic studies strongly suggest an innate origin of iconic mappings. However, dependent variables used are offline measures and especially adults' judgments might reflect metacognitive strategies.

To overcome this problem, Westbury (2005) implemented a lexical decision task in an implicit interference design. Words and nonwords matching Köhler's stimuli's consonant characteristics were presented simultaneously to either congruent or incongruent round or angular shapes. Results showed reliable form-x-phonology interaction, though for nonwords only, i.e., continuants on curvey backgrounds or plosives on angular backgrounds were rejected faster than vice versa. Therefore, sound-shape mappings appear to hold psychological reality also influencing online processing beyond judgments.

Using an implicit learning categorization task combined with EEG, Kovic et al. (2010) presented subjects with curvy or pointy figures labeled sound-symbolically congruent or incongruent as either “dom” or “shick.” After a learning phase participants had to decide whether presented label-object pairs where correct or incorrect. Responses were faster in the sound-symbolic congruent compared to the incongruent condition. Congruent sound-shape pairs further elicited an early occipital negativity around 160 ms. Based on earlier findings (Hillyard et al., 1998) the authors interpret this result as indicative of multi-sensory feature integration and covert spatial attention.

Likewise, Ramachandran points to possible synkinetic mappings of hand and jaw movements, controlled in two adjacent areas in the Penfield motor homunculus (Ramachandran and Hubbard, 2001), claiming that the “pincer-like opposition of thumb and forefinger to denote small size” might be mimicked in movements of the jaw as typically displayed in the production of front vowels (Ramachandran and Hubbard, 2001, p. 21). Contrasting high and front vowels against low and back vowels across 136 languages, Ultan (1978) suggested deictic distinctions to reflect conjoint activation of motor maps for moving of lips and hands toward and away from the body. Similarly, cross-modal mappings in the left fusiform or angular gyrus might explain non-arbitrary sound-shape correspondences via integration of visual information from the inferior temporal lobe and sound representations from the primary auditory cortex. Cross-modal associations would, then, be more likely to arise in neighboring rather than remote brain regions (Ramachandran and Hubbard, 2005) as also suggested by Bremner et al. (2012), who replicated sound-shape mappings, but failed to show reliable taste-shape mappings across distant cultures.

### Sound and affect

Building on their research on sound-size correspondences, Taylor and Taylor (1965) asked monolinguals from four unrelated languages, English, Japanese, Korean, and Tamil, to rate pseudowords comprising phonemes common to all four languages on pleasantness. Ratings showed consistent patterns within, but differed considerably across languages where different phonemes were perceived as more or less pleasant suggesting that sound-emotional meaning relations are language specific and hence likely to be learned in a given linguistic context.

Focusing on real text instead of artificial word material, Fónagy (1961) contrasted Hungarian poems characterized as either aggressive or tender. He found sonorants (e.g., /l/,/m/) to occur more often in tender but plosives (e.g., /k/,/t/) in aggressive poems. Regarding poetic text samples high in foregrounding, i.e., unexpected irregularities with regard to a common phonological inventory, Miall (2001) states that they not only display differential phonetic features, e.g., relative occurrence of front vowels and plosives, but are also perceived as more affective and striking (cf. Schrott and Jacobs, 2011). A number of cross-linguistic studies (Wiseman and van Peer, 2003; Auracher et al., 2010) following Fónagys approach corroborate parallels across remote languages like German, Chinese, Russian, Ukrainian and Brazilian Portuguese—all using non-contemporary poems.

In a more general approach, Heise (1966) extended these ideas to emotional constructs and the organization of the vocabulary. He collected valence, arousal, and potency ratings for 1000 monosyllabic English words. After segmenting words into single phonemes he found phoneme occurrences to significantly co-vary with affective scales. Extending these findings to more representative text samples, Whissell (1999, 2000) attributed phonemes' emotional quality to both place and manner of articulation as being variably related to different positions in the affective space (e.g., pleasantness, sadness, passivity, etc.).

Aryani et al.' (2013) software tool extracts a given texts' phonologically salient units, which might serve as foregrounding elements—potentially effective at a level of phonological iconicity modulating a text's emotional tone (cf. Jespersen, 1922; Schrott and Jacobs, 2011). Adopting a more acoustic approach, Myers-Schulz et al. (2013) suggested a characteristic dynamic formant shift, rather than distinct phonemes, to predict the matching of nonwords to positive or negative pictures.

Another account of systematic mappings of phonology to affective dimensions was proposed by Zajonc et al. (1989; McIntosh et al., 1997), who contrasted the umlaut /y/ with other vowels, hypothesizing that facial muscle feedback from the corrugator muscle associated with its production would cause rather negative affective states: pleasantness and mood ratings of American and German subjects became indeed more negative after the utterance of this specific vowel or after reading stories with higher occurrence of it.

## Conclusion

Systematic form-meaning mappings are abundant in many languages, although not always necessarily iconic in nature. Yet, these latter ones hold strong implications for the essence of human language and its origin.

Given the relatively small inventory of phonemes and the potentially infinite number of concepts to be expressed, the Saussureian principle of arbitrariness certainly remains a general key feature of human language (Gasser, 2004), allowing for large lexica with effective linguistic signals to develop (Monaghan et al., 2011). Nonetheless, cross-linguistic agreement and onset at early stages of language development of the outlined phenomena suggest a universal basis of motivated signs to be considered. From a phylogenetic perspective, Darwin (1871) already suggested language to originate from the imitation of natural sounds, further motivated by emotional impulse. Similarly, Ramachandran and Hubbard (2001) conjecture that language evolution might have been driven by analogies between phonology and perceptuo-motor properties of semantic entities as a solution to the symbol grounding problem (Harnad, 1990). Following Perniss and Vigliocco (in press), iconicity would thereby be essential to jump-start phylogenetic and ontogenetic development in terms of displacement and referentiality. It thus provides an additional mechanism to Hebbian learning and, regarding language processing in later stages, consequently embodies language in experience.

Fay et al. (2013) point in a similar direction, reporting that participants were able to bootstrap meaning from gesture and non-linguistic vocalization, partially depending on item category such as object, action or emotion. In analogy they argue that the evolution of signs from motivated origin to conventional use is still observable in certain sign systems such as Chinese hanzi (Vaccari and Vaccari, 1961) or American Sign Language (Frishberg, 1975).

Strictly arbitrary relations between levels of phonology and semantics as assumed by psycholinguistic models (e.g., Levelt et al., 1999) are incompatible with the effects discussed above and few promising attempts have been made to overcome respective limitations as e.g., the featural and unitary semantic space hypothesis (Vigliocco et al., 2004), or the neurocognitive poetics model of literary reading (Jacobs, 2011, 2014). More effort is thus required for future psycholinguistic theory to incorporate both arbitrariness and iconicity as essential features of human language.

### Conflict of interest statement

The authors declare that the research was conducted in the absence of any commercial or financial relationships that could be construed as a potential conflict of interest.
